# A Research on Developmental Characteristics of Children With Language Delay in Zhejiang Province, China

**DOI:** 10.3389/fped.2020.00479

**Published:** 2020-08-25

**Authors:** Dan Yao, Yan Zeng, Minjie Gao, Jiyang Shen, Jianying Zhan, Zhengyan Zhao

**Affiliations:** ^1^Department of Pediatric Health Care, The Children's Hospital, Zhejiang University School of Medicine, Hangzhou, China; ^2^National Clinical Research Center for Child Health, Hangzhou, China

**Keywords:** language delay, children, Sign-significant relations (S-S), developmental characteristics, China

## Abstract

**Study Design:** We used Sign-significant relations (S-S) to assess the developmental characteristics of 1- to 4-year-old children with language delays in Zhejiang Province and to provide scientific basis for early clinical detection and comprehensive intervention.

**Methods:** A total of 1,113 children among the ages of 1 and 4 who complained of poor language skills were assessed in language competence using S-S. These children diagnosed with language delays were divided into six groups, with each group having an age difference of 6 months. The developmental characteristics of each group were described and analyzed.

**Results:** (1) Children from the age of 18 to 36 months were most likely to be affected by language problems, while boys were more susceptible than girls in each group. (2) There was no significant difference in the proportion of children with poor communication attitude among the groups. (3) The older the group, the higher the proportion of basic learning ability abnormality. The cutoff age for qualitative leap in the proportion of basic learning abilities was 2 years old. (4) With the increase of age, the proportion of abnormal language comprehension in each group increased gradually. The cutoff age for qualitative leap in the proportion of language comprehension was 1.5 and 2 years old.

**Conclusion:** Language delays usually occur in children around the age of two, and as the children get older, in addition to expression of language abilities, they are more likely to have abnormal language comprehensive abilities and abnormal basic learning abilities. Based on the clinical research, we must take seriously the early screenings for this age group and conduct intervention training as soon as possible.

## Introduction

Language delay in young children is a very common developmental problem. The peak of this disease is around 2 years old, and the incidence rate abroad is about 15% (1, 2). Since language delay can be an indicator of several neurodevelopmental problems, it should be taken seriously. Language delay will not only seriously affect children's language comprehension and expression abilities, but also affect their social adaptability, cognitive ability, communicative ability, and other behavioral developments to a certain extent. Later in their life, children often face many difficulties in cognition, reading, writing and calculation, which seriously affect their neuropsychological development (3–5). Consequently, there is a great need for guidelines to deal with this prevalent problem (6).

Early language development in infants is an important part of children's neuropsychological development. Early detection and intervention in this stage will greatly reduce the short-term and long-term adverse effects of language dysplasia on children (7, 8). In this research, children from the ages of 1 to 4 who come to language specialists of the author's hospital with language development delay were evaluated by Sign-significant relations (S-S). This allowed doctors to analyze the comprehensive and expressive language abilities as well as non-verbal cognitive abilities of children at different ages, and to understand the developmental characteristics of children with language delays in different time periods, and provide scientific basis for the early detection and comprehensive intervention of children with language delay.

## Materials and Methods

### Research Design and Study Sample

A total of 1,113 children with language delay who sought treatment in the author's hospital between January 2018 and December 2019 were selected for this research. These children were assessed in language competence using S-S. The developmental characteristics were described and analyzed by this assessment. Language development delay was defined when the result of one's language expression ability age is lower than one's actual age or both language expression and language comprehension ability age are lower than one's actual age. After being assessed by S-S and other relevant inspections, 17 children with normal expression abilities, nine children with hearing impairment (9), seven children with mental retardation, and three children with congenital genetic diseases were excluded. This resulted in a total of 1,077 children (857 males and 220 females) with abnormal language expression abilities included for the analysis of this study. The children were divided into six groups according to their ages: 67 cases were between 1 and 1.5 years old, 346 cases were between 1.5 and 2 years old, 392 cases were between 2 and 2.5 years old, 181 cases were between 2.5 and 3 years old, 58 cases were between 3 and 3.5 years old, and 33 cases were between 3.5 and 4 years old.

### Assessment

S-S is an assessment method for diagnosing language delay, which can also describe developmental characteristics of children. The S-S method is suitable for children with Language retardation caused by various reasons from 1 to 6.5 years old. S-S was developed by the Speech Committee of the Japanese Phonetics and Speech Medical Association. This technique was standardized by the Language Department of China Rehabilitation Research Center based on the Chinese language system. The S-S results include language comprehension, language expression, basic learning ability, and attitude of communication. The ability of language comprehension usually refers to “listening comprehension.” The ability of language expression is the language symbol, which usually refers to “what to say.” Basic learning ability refers to the visual and auditory discrimination, memory, and reproduction. Communication attitude refers to the daily communication and willingness to communicate. The results of S-S should be compared with the actual age stage. If the result is lower than the actual age, the abnormal results of corresponding items can be defined.

### Statistical Analysis

The SPSS 20.0 statistical software was used to analyze the data. The data in this study were qualitative. Therefore, the chi-square test was used for comparison. The difference was statistically significant with *P* < 0.05. The difference was statistically significant with *P* < 0.05/6 between the two groups of six different ages. When pairwise comparison was conducted among multiple groups, the test level was as follows *P* < 0.05/*N* (where *N* was the required test times). There were six pairwise comparisons among the four groups in this study; therefore, *P* < 0.05/6.

## Results

### The Relationship Among Age, Gender, and Language Delay

Children with language underdevelopment problems usually started seeking treatment from 12 months old. In the early stages, cases were directly proportional to the children's ages. The trend began to decline after the age of 2.5 years old. The number of cases was highest in the 1.5–2, 2–2.5, and 2.5–3 age groups. In all the cases, boys were in the majority (857 boys and 220 girls). In each age group, boys also accounted for the majority, and the proportion of boys and girls had no difference between each group (χ^2^ = 8.726^a^, *P* = 0.120) (Table 1). Therefore, the comparison results between boys and girls in below-mentioned other projects will not be listed separately.

**Table 1:** The relationship between age, sex, and language development disorder in children (*n*, %).

	**Total**	**1–1.5**	**1.5–2**	**2–2.5**	**2.5–3**	**3–3.5**	**3.5–4**
Cases	1077	67	346	392	181	58	33
Boy	857	47 (70.1%)	275 (79.5%)	311 (79.3%)	144 (79.5%)	49 (84.5%)	31 (93.9%)
Girl	220	20 (29.9%)	71 (20.5%)	81 (20.7%)	31 (20.5)	9 (15.5%)	2 (6.1%)

### S-S Was Used to Evaluate the Performance Characteristics of Each Item in Different Age Groups (Table 2)

According to the distribution of various ability characteristics of each group, the percentage of abnormal language expression in each group was 100%, all the children in the study group had abnormal language expression abilities. Hence, it was not necessary to compare the differences among the six groups.

**Table 2:** Communication attitudes, basic learning ability, language comprehension ability, and language expression ability among each age group.

	**Communication attitudes**		**Basic learning ability**		**Language comprehension ability**		**Language expression ability**	
	**Good**	**Poor**	**Normal**	**Abnormal**	**Normal**	**Abnormal**	**Normal**	**Abnormal**
Total	935 (86.8%)	142 (13.2%)	415 (38.5%)	662 (61.5%)	130 (12.1%)	947 (87.9%)	0 (0%)	1077 (100%)
1–1.5	61 (91.0%)	6 (0.9%)	50 (74.6%)	17 (25.4%)	25 (37.3%)	42 (62.7%)	0 (0%)	67 (100%)
1.5–2	300 (86.7%)	46 (13.3%)	201 (58.0%)	145 (42.0%)	71 (20.5%)	275 (79.5%)	0 (0%)	346 (100%)
2–2.5	348 (88.8%)	44 (11.2%)	114 (29.1%)	278 (70.9%)	25 (6.4%)	367 (93.6%)	0 (0%)	392 (100%)
2.5–3	147 (81.2%)	34 (18.8%)	36 (19.9%)	145 (80.1%)	6 (3.3%)	175 (96.7%)	0 (0%)	181 (100%)
3–3.5	48 (82.7%)	10 (17.8%)	12 (20.1%)	46 (79.9%)	2 (3.4%)	56 (96.6%)	0 (0%)	58 (100%)
3.5–4	31 (93.9%)	2 (6.1%)	2 (6.1%)	31 (93.9%)	1 (3.0%)	32 (97.0%)	0 (0%)	33 (100%)

**Figure 1:**
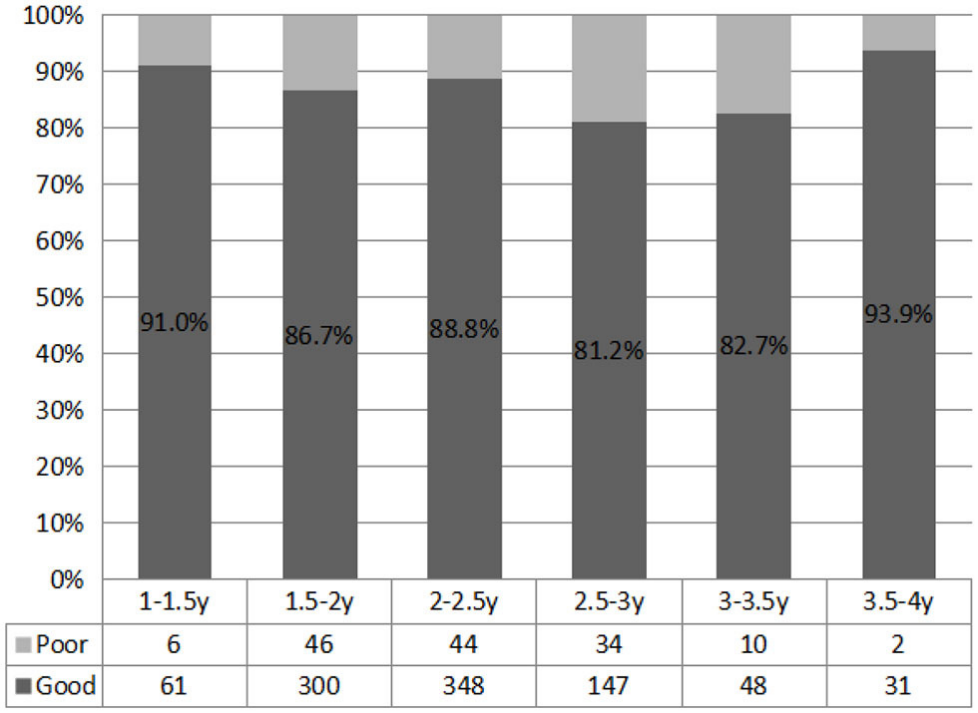
Difference in communication attitudes among different age groups (*n*, %).

The second most common abnormality was language comprehension in each group. There were differences in the proportion of children with abnormal language comprehension among the six groups (χ^2^ = 95.147^a^, *P* = 0.000). The data showed that with the increase of age, the proportion of abnormal language comprehension in the group increased gradually. Through the comparison between the two adjacent groups, it was found that the most significant difference between components was the 1- to 1.5-year-old group and the 1.5- to 2-year-old group, the 1.5- to 2-year-old group, and the 2- to 2.5-year-old group (χ^2^ = 8.872^a^, *P* = 0.003; χ^2^ = 32.484^a^, *P* = 0.000). The difference was statistically significant. It can be seen that the cutoff age of qualitative leap in the proportion of people with abnormal language comprehension ability is 1.5 and 2 years old (Figure 1).

**Figure 2:**
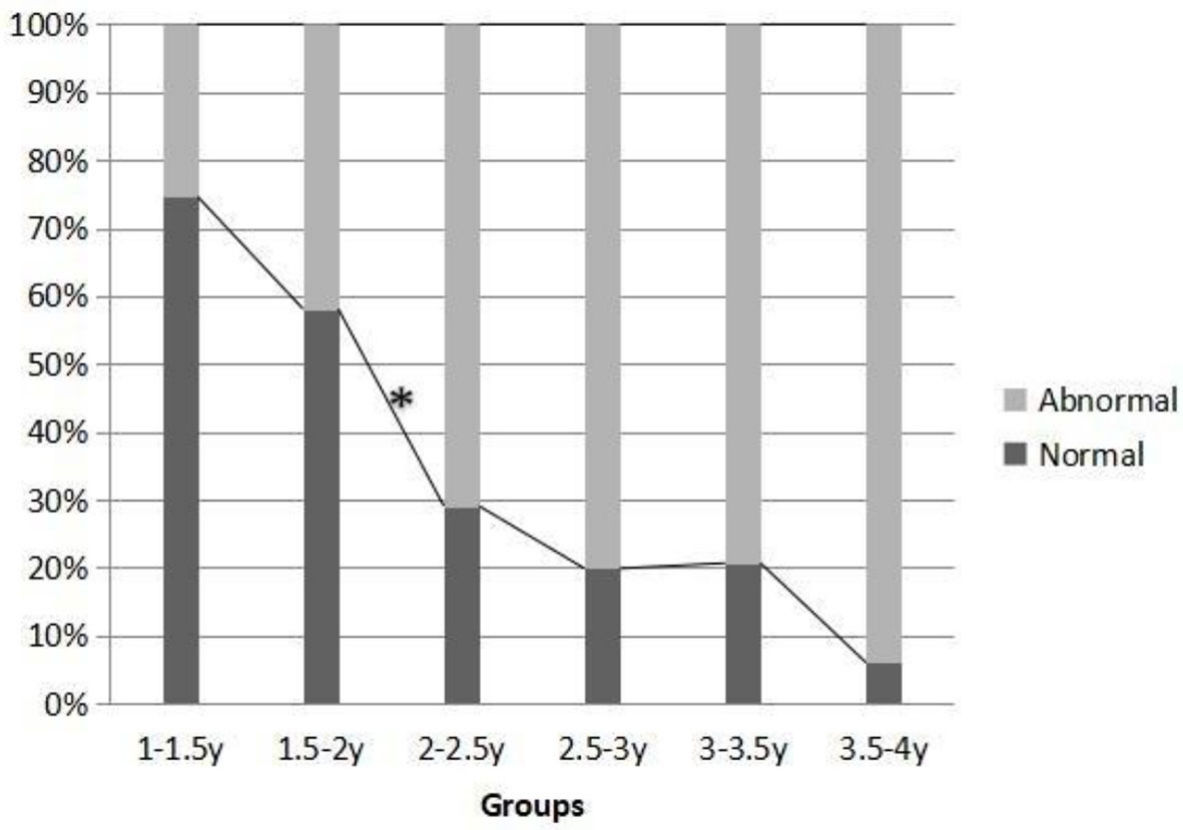
Difference in basic learning ability among different age groups (*n*, %). *There were significant differences between the representative groups.

The third highest proportion of abnormality was abnormal basic learning abilities in each group. There were differences in the proportion of children with abnormal basic learning abilities among the six groups (χ^2^ = 156.575^a^, *P* = 0.000). The data showed that with the increase of age, the proportion of basic learning ability abnormalities increased. From the beginning, the number of normal basic learning abilities in each group was more than that of the abnormal ones, but after the age of two, there was a reversal. The number of abnormal basic learning abilities was more than that of the normal ones, and the gap was growing. Through the comparison between the two adjacent groups, it was found that the most significant difference between components was the 1.5- to 2-year-old group and the 2- to 2.5-year-old group (χ^2^ = 63.23^a^, *P* = 0.000). The difference was statistically significant. It can be seen that the cutoff age of qualitative leap in the proportion of abnormal action subjects is 2 years old (Figure 2).

**Figure 3:**
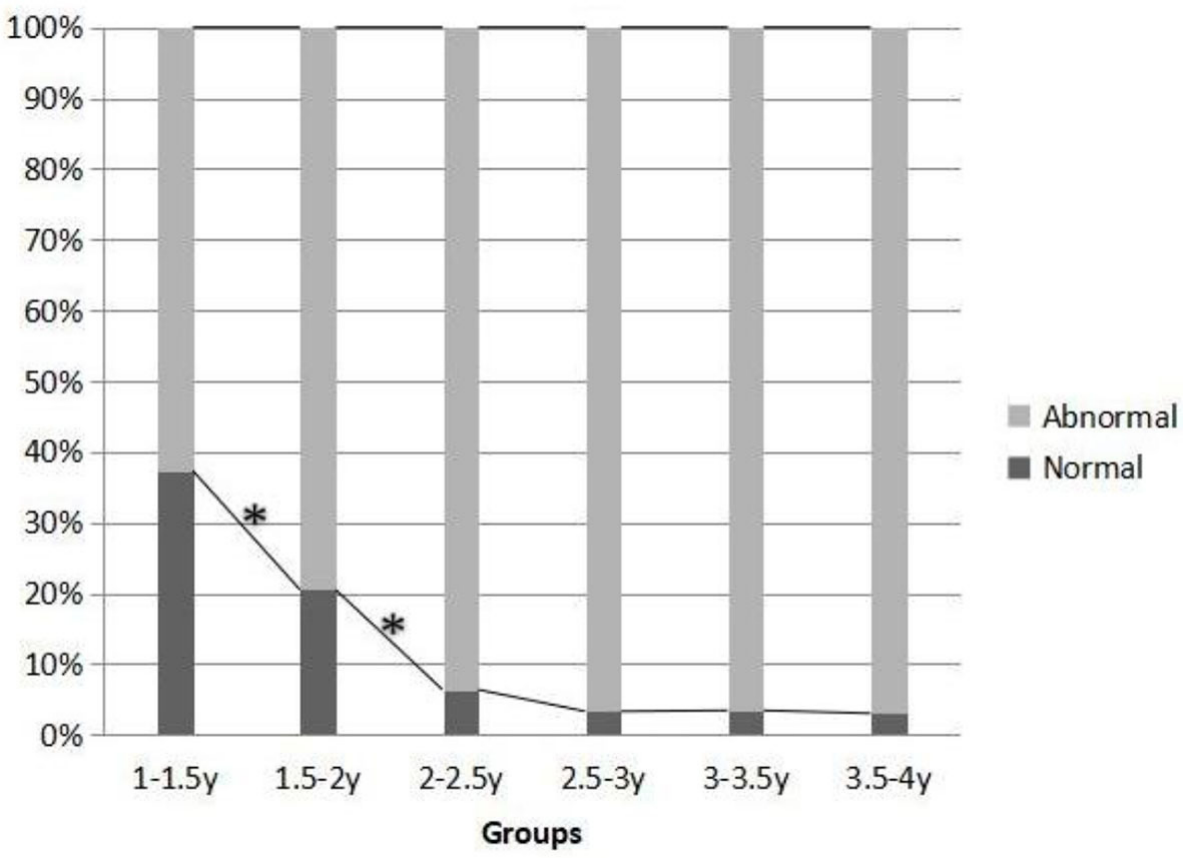
Difference in language comprehension among different age groups (*n*, %). *There were significant differences between the representative groups.

In each group, the most abnormal communication was in the minority. Each age group had a different proportion of poor communicators. However, there was no specific difference in the proportion of poor and good communication attitudes between each group (χ^2^ = 9.622^a^, *P* = 0.087) (Figure 3).

## Discussion

### The Relationship Among Age, Gender, and Children's Language Delay

Language delay, which is a common developmental problem, is most prominent in children of 2 years old. Children's congenital factors and acquired language environment will both affect the occurrence and development of language delay and have a certain impact on their neuropsychological development (10). The first 3 years after birth are the critical period for children's brain development. The critical period for the development of intuitive action thinking to specific image thinking is also 2–3 years old. Therefore, this is also an important period for children's oral language development and social interaction learning (11). This study shows that the incidence of language delay in children around the age of two is the highest, which is consistent with the highest incidence in foreign countries (1, 2, 12). At the same time, most of the children with language delay, regardless of their age, are boys, which is also consistent with the foreign studies (13). Male children have a high incidence of language delay, and the reasons may be due to the slow maturation of the central nervous system in boys and the influence of testosterone (14). Testosterone can prevent cell death and make it difficult to connect the appropriate language center (15). Although there was no significant difference among the six age groups, the proportion of boys in the 1- to 1.5-year-old group was lower than that in the other five groups. It may be because that language lag may also occur in girls between 1 and 1.5 years of age, but in the later stage, girl's language ability will be improved faster than boys. A small part among them can catch up with the normal level, so the gap between boys and girls in the later stage is obvious (16).

### The Developmental Characteristics of Children in Different Ages From S-S

Language delay includes two parts: language comprehension and language expression. S-S can evaluate the language development level of children over 1 year old. It mainly evaluates the language development of children from four aspects: basic learning ability, comprehension ability, expression ability, and communication attitude. It can reflect the differences of children's language comprehension and expression, as well as the evaluation and observation of basic learning ability and communication attitude.

All the participants in this study were accompanied by abnormal language expression, so abnormal language expression ability was not analyzed separately. There is a certain proportion of poor communication attitude in each age group, but there is no obvious correlation with age. As they get older, the proportion of children with language delays with abnormal basic learning abilities and comprehension abilities increases gradually, and the proportion of children with abnormal basic learning abilities reversed after the age of two, which is the cutoff age of qualitative leap. At the same time, the cutoff age of qualitative leap in the proportion of people with abnormal language comprehension abilities is 1.5 and 2 years old. It can be seen that the younger the age is, the higher proportion the pure abnormal language expression is. However, after the age of two, the possibility of abnormal language expression combining with other problems peaks, and it is more likely to be complicated with other developmental disorders (17). All of these suggest that both families and hospitals should understand the development characteristics of children with language delay in different age groups and correctly establish an awareness of prevention, so that we can detect children with language delay in the early stage, and do a comprehensive intervention to avoid delaying treatment (12, 18, 19).

However, some limitations should be noted. First, references related to the S-S are scarce, so the theoretical basis of the research will be relatively inadequate. However, on the other hand, this provided a new direction of exploration. Second, S-S is not only an evaluation method, so it can also be used to guide the training according to the results. In addition to a horizontal description, we can also conduct a vertical cohort study, so that our next step is to evaluate the changes in the developmental characteristics of the child after the assessment and intervention by S-S, in order to further illustrate the usefulness of this method.

Clinical data show that the incidence of language delay in children is not low, and there is a growing trend with the development of modern media. The critical time to discover language delay is when the child is around 2 years old, but children's early language development problems do not receive proper attention due to the traditional concept of “the noble speaks later” and the normal comprehensive ability of some children in early childhood (20, 21). At the same time, most parents are not sensitive enough to children's language comprehensive abilities and not attentive enough to language expression abilities, so they may not be able to detect and intervene early (22, 23). In this regard, attention should be paid to the characteristics of language development and intelligent development of infants around 2 years old. Medical staff should properly educate parents on the health of their children; they should also introduce the stages, characteristics, and poor performance characteristics of children's language ability development (24), so that parents can detect the child's language delay in the first place and seek professional help and receive relevant intervention treatment as soon as possible, thus reducing the incidence of language delay with other developmental disorders.

## Data Availability Statement

All datasets generated for this study are included in the article/supplementary material.

## Ethics Statement

The studies involving human participants were reviewed and approved by The Children's Hospital, Zhejiang University School of Medicine and Affiliation of Ethics Committee. Written informed consent to participate in this study was provided by the participants' legal guardian/next of kin.

## Author Contributions

DY: designed the study, enrolled patients, analyzed the data, and drafted the manuscript. YZ: enrolled patients, collected, and analyzed the data. MG and JS: did the Sign-significant reations and entered data. JZ: enrolled patients, supervised data collection, and analysis. ZZ: conceived the study and critically revised the manuscript. All authors contributed to the article and approved the submitted version.

## Conflict of Interest

The authors declare that the research was conducted in the absence of any commercial or financial relationships that could be construed as a potential conflict of interest.
